# Electrical Impedance Tomography: From the Traditional Design to the Novel Frontier of Wearables

**DOI:** 10.3390/s23031182

**Published:** 2023-01-20

**Authors:** Francesca Pennati, Alessandra Angelucci, Letizia Morelli, Susanna Bardini, Elena Barzanti, Federico Cavallini, Antonello Conelli, Gaia Di Federico, Chiara Paganelli, Andrea Aliverti

**Affiliations:** Dipartimento di Elettronica, Informazione e Bioingegneria, Politecnico di Milano, 20133 Milan, Italy

**Keywords:** electrical impedance tomography, wearable, imaging

## Abstract

Electrical impedance tomography (EIT) is a medical imaging technique based on the injection of a current or voltage pattern through electrodes on the skin of the patient, and on the reconstruction of the internal conductivity distribution from the voltages collected by the electrodes. Compared to other imaging techniques, EIT shows significant advantages: it does not use ionizing radiation, is non-invasive and is characterized by high temporal resolution. Moreover, its low cost and high portability make it suitable for real-time, bedside monitoring. However, EIT is also characterized by some technical limitations that cause poor spatial resolution. The possibility to design wearable devices based on EIT has recently given a boost to this technology. In this paper we reviewed EIT physical principles, hardware design and major clinical applications, from the classical to a wearable setup. A wireless and wearable EIT system seems a promising frontier of this technology, as it can both facilitate making clinical measurements and open novel scenarios to EIT systems, such as home monitoring.

## 1. Introduction

Electrical impedance tomography (EIT) is a radiation-free, real-time imaging technique which reconstructs the internal conductivity distribution of a subject based on electrical stimulation and measurements on the body surface. Although the traditional name was agreed on a long time ago at the first Sheffield meeting in 1986, it is not a proper tomographic technique. Indeed, it is not possible to reconstruct an image slice by slice because low-frequency electrical current cannot stay confined in a plane: a change in conductivity anywhere in the domain affects all measurements, not only those on the specific slice [1].

As a medical imaging modality, EIT has several advantages. It does not use ionizing radiation, is non-invasive, is characterized by high temporal resolution and is potentially inexpensive. Its major limitations include lower spatial resolution compared to other imaging techniques (such as echography, magnetic resonance imaging, and computed tomography), the large inter-subject variability and the artifacts derived from the electrodes’ movement and poor contact quality. Different hardware solutions [2,3] and reconstruction algorithms [4,5] have been proposed to improve image resolution. Although EIT still cannot compete with the high spatial resolution and accuracy of other imaging techniques, it has significant potential to be used in parallel to overcome many of their limitations. Moreover, the possibility to design wearable devices based on EIT has recently given a boost to this technology, which may represent a unique wearable imaging technique.

Numerous review papers have been published on EIT, including general overviews [6], reviews of image reconstruction algorithms [7] and works on clinical applications [8]. This review paper is aimed to give a new viewpoint on this technology, focusing on the hardware design developments from classical systems to the most promising wearable implementations. First, the electrical properties of tissues are described, followed by the presentation of the classical set-up of an EIT system and the main fields of application, with a focus on the design choices for the electrodes, the stimulation strategy and the hardware implementation. Then, the main features requested by wearable EIT technology are discussed, presenting the most relevant and recent wearable devices reported in the literature.

### Bioimpedance

Bioimpedance is described as the ability to impede electric current by a biological tissue [9]. Generally, bioimpedance is a complex number composed of a real part (the electric resistance, *R*) and an imaginary part (the capacitive reactance, *X_C_*):

$$Z=R+jX_{C};$$

The electrical properties of biological tissues have been widely studied in the literature [10], even in relation with physiological phenomena [11]. Biological tissues consist of cells suspended in extracellular fluid. Each cell has a membrane enveloping intracellular fluid. Both the intracellular and extracellular fluids are highly conductive and provide resistive paths. The lipid bilayer cell membrane, which separates the intracellular and extracellular fluids, acts as a dielectric and provides capacitive reactance.

The electrical properties of the tissues depend on the frequency of the applied electric field. At low frequencies, the membrane impedance is high, and the current mainly flows through the extracellular space. As the current flows around the cells, the electrical path length is high, increasing the resistance of the medium. At higher frequencies, the impedance of the membrane reduces, and the current flows through both the extra- and intracellular media. As the cross-sectional area through which the current flows is increased, the effective electrical path length lowers, decreasing the impedance of the medium. Through most frequency ranges, the impedance decreases with frequency, but the curve is interspersed by plateaus called dielectric dispersions [12].

The specific composition of human tissues determines their bioimpedance, which ranges from around hundreds of Ω to hundreds of kΩ. High content of extracellular water, high concentration of electrolytes, large cells and a high number of cell connections reduce impedance, whilst fat accumulation, bone and air increase impedance [13]. Therefore, changes in the tissue composition, both physiological and pathological, entail a change in regional bioimpedance [14]. Electrical properties of tissues may also be influenced by other factors, which must be considered when measuring tissue impedance, such as temperature, chemical and pH changes, neural and muscular activation and anisotropy dependance [15,16].

## 2. Electrical Impedance Tomography

An EIT system reconstructs the bioimpedance map of a conducting domain from the surface potential, created by injecting a low-intensity alternate current at the object boundary. The system design requires the following steps:

- Choose the imaging modality (absolute or differential) and define the reference dataset for the differential approach.
- Select suitable electrodes (number, position and material).
- Define the measurement strategy (voltage-mode or current-mode).
- Design the electronic hardware, including both the current generation and the voltage readout blocks.
- Choose the numerical algorithm to solve the inverse problem.

The EIT problem that reconstructs the impedance distribution starting from the applied current patterns and the measured electrode voltages, the so-called inverse problem, is challenging. Compared to X-ray computed tomography, where the photons paths are straight lines, in EIT the current flow is determined by the impedance distribution within the object, and thus image reconstruction is a highly nonlocal and mathematically ill-posed problem [1]. It should be highlighted that details of EIT reconstruction algorithms are out of the scope of the present review. Possible solutions are widely discussed in the literature [7,17], also with some techniques to increase robustness [18].

### 2.1. EIT Imaging Modality

Two EIT imaging modalities can be employed: absolute and differential [19].

Absolute EIT uses a single experimental dataset to reconstruct a map of the absolute impedance. Despite its theoretical applicability, in clinical applications unknown boundary geometry, uncertainty in electrode positions and other sources of systematic artifacts make image reconstruction unreliable [20].

In differential EIT, instead, the image is obtained by measuring two datasets at different times (time-difference EIT) or frequencies (frequency-difference EIT) and measuring the impedance changes between the two datasets. This technique is more robust against instrumentation and modelling errors [21].

Time-difference EIT is well suited to monitor time-varying physiological phenomena, but is applicable only to specific application cases where time variation occurs, such as lung ventilation and perfusion. On the contrary, frequency-difference EIT measures the different impedance characteristics of tissues at different measurement frequencies, allowing image reconstruction when time-referenced data are not available. Nevertheless, its sensitivity depends on the difference between electrical frequency properties of the tissues, which may be insufficient for some application cases [22,23]. The different EIT imaging modalities and their advantages and disadvantages are listed in Table 1.

### 2.2. Measurement Strategies

There are two possible approaches for signal acquisition: (1) voltage-mode EIT, in which a known voltage is applied to the tissue, and the induced currents are measured; (2) and current-mode EIT, in which a known current is injected, and the resulting voltage generated on the body surface is measured. Voltage-mode EIT systems are easier and less costly to design and have a stable response over a wide frequency range [24]. Nevertheless, current-mode EIT systems are generally preferred, as they are less sensitive to noise and it is easier to limit the maximum current injectable to guarantee the safety of the system [24].

Current injection follows different strategies, according to the application:

- Adjacent method: Current is injected between two adjacent electrodes, and voltage is acquired between all the remaining pairs of adjacent electrodes. This is the most common technique [25].
- Opposite or Polar method: Current is injected between two electrodes that are 180° apart, and voltage is measured between the reference electrode and the remaining electrodes in pairs. This technique is used in the brain-imaging field [26].
- Cross or Diagonal method: One electrode is kept as a reference for the voltage measure, and current is injected between all the possible pairs of electrodes in turn; the measure is repeated by taking one electrode as a reference each time until all are used. This method is not usually used in EIT [26].
- Trigonometric method: Current is injected through all the electrodes at the same time instant, and the voltage is measured with respect to a reference electrode. This is the technique that guarantees the highest number of independent measures [25].

The frequency of the injected current depends on the conductivity and permittivity of the tissue under analysis [27]. The standard measurement frequency is 50 kHz, but some devices analyze multiple frequencies (10 Hz–10 MHz) to improve the image quality and to analyze different tissues. Frequencies higher than 1 MHz, however, are challenging, primarily due to parasitic impedances that corrupt the signals; thus, specific high-performance circuit components and data acquisition modules are required [24,28].

### 2.3. Electrodes

The electrode system represents the electrochemical interface between the electronic system and the body tissue, and it represents the most critical part of a bioimpedance measurement system.

#### 2.3.1. Number

The first open problem is the number of electrodes to be used. Increasing the number of electrodes increases image resolution, as more independent measurements are available, but decreases precision and requires greater measurement and processing time [18]. Moreover, increasing the number of electrodes increases contact impedance [25]. The most common solutions employ 16 electrodes, but innovative configurations with up to 90 electrodes have been proposed for cancer detection [29]. A higher number of electrodes is required in 3D systems, ranging from 48 to 128 electrodes for lung imaging applications [30,31]. To increase the number of possible independent measurements without increasing the number of electrodes, rotational EIT has been proposed: the electrodes are rotated to acquire additional data. The rotation is performed with a micro stepping motor [32] to precisely change the positions of the electrodes. Promising results have been achieved [33], particularly in breast cancer detection [34].

#### 2.3.2. Positioning

The position of the electrodes on the boundaries is also critical for image reconstruction algorithms and may cause lack of robustness [18]. It is quite difficult to achieve standard placement on the body surface [35], often due to the need for individual application of electrodes. This problem is even enhanced in wearable devices where a system could be applied or at least re-collocated by non-specialized personnel. To overcome this problem, a belt solution greatly reduces both the time dedicated to manual positioning and the inter-operator variability [25]. In addition, an elastic or adjustable electrode belt may even solve the issue of adapting a wearable system to different body sizes [36].

#### 2.3.3. Contact Impedance

Contact impedance is also an issue of concern in EIT. Contact impedance is caused by the electrochemical process occurring at the electrode–skin interface, in which the charge carriers, responsible for the current flow, change at the interface between the metallic electrode and the body surface, causing a drop to the measured voltage at each electrode. Contact impedance is usually high, unknown and variable due to body movement and surface conditions [35]. It causes modelling errors and, if not compensated for or estimated, leads to errors in the reconstructed image [37]. To reduce the effect of contact impedance, a multi-pole measurement strategy with four or more electrodes can be adopted, which provides a higher impedance of the measuring device compared to contact impedances [38]. Another approach to reduce contact impedances is to treat them as unknowns and simultaneously reconstruct them with internal electrical properties [39]. Also, the capacitively coupled EIT technique (CCEIT), which induces a voltage excitation through a capacitive coupling, has been recently introduced as a contactless solution [40].

#### 2.3.4. Interference Sources

A high contact impedance also means that all interference sources, such as common mode gain error, thermal noise and electromagnetic interference and crosstalk between cables, degrade image resolution [35]. To solve this issue, active electrode-based EIT systems have been proposed, in which each active electrode comes with its own signal conditioning circuit, thus reducing input impedance. Other than reducing input impedance, this architecture has several advantages, including less sensitivity to electromagnetic interferences and a reduced number of cables needed from the patient to main electronics, leading to a more comfortable system [30,35,41,42,43,44].

#### 2.3.5. Contact

In biomedical applications, ensuring good contact is also critical because the electrodes are often placed on a deformable tissue [18]. Adhesive Ag/AgCl electrodes with conductive gel are commonly used. A safer contact may be guaranteed with volume electrodes, but they are invasive and size-constrained to limit the skin damage due to injected current [18]. A faulty contact detector algorithm [45] could also be used: despite partial detachments, a good image reconstruction could be guaranteed, and the identification of faulty electrodes would allow personnel to fix them.

#### 2.3.6. Requirements for Wearable EIT

A specific requirement for wearable applications is to ensure a good contact during motion and for long-term use. In fact, wearable devices are suitable for prolonged monitoring of physiological parameters because they are not invasive and generally wireless. Wearables are especially useful in monitoring chronic conditions such as COPD or heart failure; therefore, a long term, independent use in a non-controlled environment (such as the patient’s home) is envisioned. Ag/AgCl electrodes with conductive gel may be problematic for these applications: the preparation of the patient’s skin may be problematic in a non-controlled environment [46]; the conductive gel may cause discomfort, increasing the risk of skin injury for prolonged measurements and may evaporate, resulting in a deterioration of the signal quality [47]. So, dry electrodes (not requiring a conductive gel) are usually preferred for wearable devices [46], both rigid (made of a metallic plate) and flexible. Flexible electrodes may overcome the adherence issue during motion. For this reason, they are the most commonly used variety, usually mounted on an elastic and adjustable belt [36,46,47,48]. Fabric-based flexible electrodes’ characteristics can be shortly summarized: they should be made of a textile material with high conductivity; a cotton-based fabric should be preferred for higher comfort; the textile electrode should be a fixed part of the clothes for easier system application and stability; and the textile material should allow general maintenance such as washing and ironing [47]. Textile electrode belts with good performances have been broadly proposed in the literature [47,48]. Dry and flexible electrodes were also proposed for brain EIT by Lin et al. [36]. Their solution is based on a dry foam fabricated by electrically conductive polymer foam covered by a conductive fabric which also allows a high level of geometric conformity between the electrode and irregular scalp surface to maintain low skin–electrode interface impedance, even under motion. In general, these novel solutions for electrodes show the same performances as the standard Ag/AgCl, but they provide a better usability for wearable devices.

An overview of the issues, challenges and innovative solutions that are found in the choice and design of the electrodes is reported in Table 2.

### 2.4. Hardware Design

**Figure 1:**
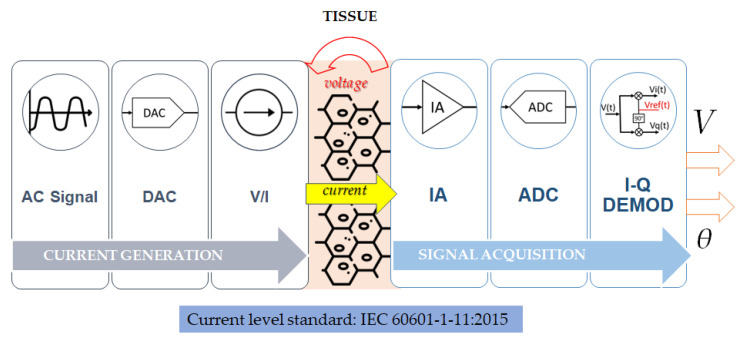
Block diagram of the hardware components of an EIT signal acquisition platform. The current generation block includes the digital signal generator, the digital to analog converter (DAC) and the voltage-to-current converter (V/I). The current is injected into the tissue, and the voltage generated on the surface is acquired by the signal acquisition block, where the instrumentation amplifier (IA), the ADC and the demodulator are found. The amplitude and the phase of the registered voltage are processed, and the impedance distribution is reconstructed.

In Figure 1, the block diagram of the hardware components is represented. The current injection block comprehends the digital signal generator, the DAC, which converts the digital signal into an analog signal, and the voltage-to-current converter. The excitation current is injected into the tissue according to the chosen strategy, and the voltage generated on the surface is acquired by the data acquisition block, where the instrumentation amplifier, ADC and demodulator are found. The amplitude and phase of the registered voltage are processed, and the impedance distribution is reconstructed.

#### 2.4.1. Current Generation and Injection

Current injection can be performed by using a DAC-based open-drain current mirror or a voltage-to-current converter [41]. The current mirror is a circuit that replicates the current present in the input terminal on the output terminal. The digital-to-analog converter (DAC) is used to convert the digital signal into the analog one, which is then used to generate the excitation current. The voltage-to-current converter uses the voltage as input, from the DAC, to produce the output current; this circuit can be implemented with a transconductance amplifier [27]. The waveforms used for the signal generation are square waves or pseudo sine waves [49]: the square wave has good power efficiency but limited accuracy, while the pseudo sine has both power efficiency and accuracy but less total harmonic distortion (THD) than a pure sine wave. The total input current level over all electrodes is limited by electrical safety considerations, as defined by the IEC 60601-1-11:2015 standard [50]:

$$I=\{\begin{array}{c} 100\mu A_{rms}\;0.1\;Hz<f<1\;kHz\\ 100\mu A_{rms}\times \frac{f}{1\;kHz}\;1\;kHz<f<100\;kHz\\ 10\;mA_{rms}\;f>100\;kHz \end{array}$$

For this application, the current must be fully differential [51]: the current at the source of the current driver must be equal to the current at the sink. In this configuration, the voltage measured is the differential voltage generated on the load by the injection of the current. A current mismatch between the source and sink of the driver generates a common-mode voltage that can either saturate the output of the current driver or generate a common-mode signal that must be rejected by the instrumentation amplifier in the subsequent steps of the voltage acquisition block [52]. To reduce the common-mode voltage amplitude, a frequency-selective common mode feedback is described in [53]. In [52], the issue is mitigated by the implementation of a fully differential current driver with a built-in common mode rejection system by using an active feedback current sink.

Recent literature proposes modifications of this basic configuration to achieve specific goals. In [54] a buffer-mirrored current source is proposed: it ensures a wide-range constant current and a high SNR. To produce an adjustable current with different frequencies and amplitudes, a mirror circuit with an input current generated by an FPGA is used in [55]. An FPGA is also used in [56] to perform the acquisition with a high sampling rate and accuracy for real-time imaging. In [27], an FPGA is used to store the digital signal in the ROM, which is sent at fixed time intervals to the DAC to convert it into an analog excitation voltage. In [57], a new current driver is presented, and it includes a servo loop to adjust the DC output voltage.

#### 2.4.2. Voltage Signal Acquisition

The voltage signal acquisition circuit for EIT in biomedical applications must be characterized by high precision, low noise, wide dynamic range, wide bandwidth and a high common-mode rejection ratio [58].

The instrumentation amplifier can be coupled with a programmable gain amplifier, or PGA, due to the presence of ‘U’-shaped voltages: the amplitude of the voltage signal acquired decreases with the distance from the injecting electrodes, generating a peculiar pattern of voltage amplitude [41]. By considering the large difference in amplitude of the different measures of all the electrode pairs, a PGA can adjust its gain accordingly. In [59], a pre-amplifier constituted by a low-noise instrumentation amplifier is used before the PGA, resulting in a possible amplification that goes from 4 to 514 times the input signal.

In [29], the PGA is coupled with an automatic gain controller (AGC) that dynamically adjusts the gain of the PGA according to the input amplitude. This is fundamental to acquire signals in a large dynamic range.

In [60], the PGA is preceded by a unit gain amplifier to increase the input impedance and is digitally controlled by the DSP, with a maximum achievable gain of 8000 times the input signal; the acquisition system is capable of considering the boundary voltage to ensure data accuracy.

The most used ADC type is the SAR ADC [61]: the sample rate must be in the mega samples-per-second range to achieve the required frame rate for the imaging system.

I-Q demodulation is the most common method used in the demodulation of bioimpedance measurements: the aim is extracting the amplitude and phase of the acquired AC voltage signal by multiplying the signal with a reference signal to obtain the in-phase component and using a reference signal shifted by 90° to obtain the quadrature phase component [41].

The in-phase and quadrature phase signals can be analog signals, which are then filtered by the low-pass filter and converted with an ADC; in this case we have an analog matched filter. Alternatively, the in-phase and quadrature phase signals can be retrieved from the digitalized original signal; in this case we have a digital matched filter. A digital filter is more power-efficient than an analog one with the same SNR [24].

The most common demodulation methods are magnitude and phase detection, pulse width demodulation and time stamp demodulation [41].

## 3. Applications

Image resolution largely depends on the EIT hardware, including the noise of the measurements, the number of electrodes, the current source and the voltage measurement techniques. Thus, the spatial resolution of the image varies from one device to another.

### 3.1. Pulmonary Imaging

Two main physiological processes contribute to thoracic bioimpedance change: ventilation and perfusion. The change in the amount of air during ventilation is responsible for a bioimpedance change proportional to the volume of inspired gas. During quiet breathing, lung tissue impedance changes by around 5%, while in deep breathing the change can reach up to 300% [62]. On the other hand, pulmonary perfusion leads to impedance changes between systole and diastole of about 3% [63]. Temporal resolution achieves 13 frames/s, with the potential to cover both ventilation and perfusion in real time [64]. The spatial resolution of the images is significantly lower and limited to the distance between electrodes (about 2–3 cm). Approximately 5 cm of lung height is sampled with the classical 16-electrode array configuration.

**Figure 2:**
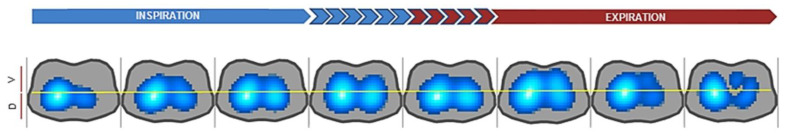
Sequence of electrical impedance tomography images, representing the distribution of ventilation in the ventral (V) and dorsal (D) regions during quiet breathing (Enlight 1800, Timpel SA, San Paulo, Brazil). The gradation of color from lighter to darker represents, respectively, the highest and lowest electrical impedance (higher and lower air displacement during the cycle). Courtesy of Caio CA Morais and Armele Dornelas de Andrade (Physiotherapy Department, Universidade Federal de Pernambuco, Recife, Brazil).

Ventilation EIT imaging has been validated using established imaging techniques [64,65,66] and other reference methods, such as spirometry [67,68]. Representative EIT images showing the distribution of ventilation during quiet breathing are reported in Figure 2. Monitoring of mechanical ventilation is the most promising application of ventilation EIT, as it provides real-time information on ventilation distribution at bedside, with no need to transfer the patient and supportive equipment. Studies have been performed for guiding ventilator settings to prevent ventilator-induced lung injury [69,70,71] and for monitoring non-conventional modes of mechanical ventilation [72]. Moreover, EIT may be used to identify adverse events, such as pneumothorax or derecruitment, during mechanical ventilation [73], enabling early therapeutic intervention. Studies have shown that EIT measurements are also feasible in preterm infants [74], and the technique has been applied to monitor therapies [75] and interventions [76].

Recent studies have focused on the use of EIT to estimate regional pulmonary perfusion. The measurement of impedance pulsatility during the cardiac cycle, based on electrocardiography gating or principal component analysis algorithms, represents the principal method for assessing perfusion by EIT [77]. Another option, described and validated only in experimental studies, is to inject an intravenous saline bolus and analyze the tracer kinetics [78]. EIT perfusion imaging has demonstrated a high degree of agreement with the gold standard represented by multidetector computer tomography (MDCT) to compute regional pulmonary perfusion [78]. With the recent outbreak of severe acute respiratory syndrome coronavirus-2 (SARS-CoV-2), several studies have been conducted with EIT imaging, showing its potential to optimize clinical ventilation strategies [79,80].

One of the main issues in the process of regional lung function imaging is defining appropriate regions of interest (ROI) within the EIT scans. To this aim, a number of different algorithms for lung area estimation have been explored, ranging from principal component analysis [77] to ROI definition based on statistical methods or geometrical considerations [81] to functional tidal images or active contouring methods [82].

### 3.2. Brain Imaging

As the blood is characterized by lower impedance compared to the brain, brain imaging exploits the impedance changes in the cerebral cortex induced by a change in the cerebral blood volume during functional activity or in the case of stroke and ischemia [83]. EIT has broad possibilities in diagnosis and real-time monitoring in patients with brain diseases, with recent research focusing on epilepsy, stroke, brain injury and edema [83]. Studies on conductive changes in the rat cerebral cortex during physiological evoked activity [84] and epileptic discharges [84] using epicortical electrodes have reported temporal and spatial resolutions of 2 ms and 200 μm, respectively. Using epicortical electrodes, a penetration depth of 3 mm has been reported for imaging epileptic activity in subcortical structures [85]. An interesting application for the unique real-time and portable characteristics of EIT is early and rapid detection of stroke, which is not achievable with current imaging techniques. A recent study reported that cerebral ischemia in anesthetized rats lead to an increase in impedance of about 60%, with a detected impedance increase of about 10–20% at the cortical electrodes [86]. 

In brain imaging settings, capacitively coupled EIT (CCEIT) is currently being investigated as a promising alternative to the traditional EIT technique [40]. Practical experiments carried out with a 12-electrode CCEIT device on phantom, saline and carrot samples showed the potentiality and feasibility of CCEIT for stroke imaging, detecting anomalies of about 10 mm of diameter located 30 mm from the boundary [40]. EIT feasibility has also been investigated for the detection of small bleeds (volumes of about 5 mL) in the brain [87] and for real-time monitoring of cerebral edema [88]. In brain imaging, image reconstruction is extremely challenging, as the skull is highly resistive, and the image quality is sometimes not sufficient, for example, when the space between the hemorrhage and ischemia is too small. An innovative solution is to use superimposed imaging for simultaneous reconstruction in order to obtain more accurate images characterized by a higher contrast [89]. This can be accomplished by superimposing the reconstructed image of the hemorrhage against the homogeneous distribution, with the reconstructed image of the secondary ischemia against the hemorrhage [89].

Although there are several issues to be addressed, EIT technology could be an effective complement to conventional imaging methods in the diagnosis and monitoring of cerebrovascular disease, enabling early detection of intracranial pathological changes and providing useful tools to improve patient prognosis [83].

### 3.3. Thermal Monitoring

Hyperthermia treatment is a minimally invasive and effective option in treating solid tumors, such as hepatocellular carcinoma [90]. Radio-frequency ablation (RFA) and microwave thermal ablation (MTA) are examples of techniques employed for the treatment of solid tumors. These approaches are considered potentially effective, being minimally invasive and safer compared to other techniques [90,91]. The principle of operation of this treatment approach is based on the conversion of electromagnetic energy into heat to cause the denaturation of intracellular proteins and cell destruction. Specifically, to induce cell destruction, the temperature is raised to around 45–50 °C, while remaining safely away from temperatures that could induce adverse effects such as tissue carbonization at temperatures above 90 °C. For this reason, the RFA probe must necessarily include a cooling system as well. The critical requirement of these techniques is to ensure the selectivity of treatment [90]. Thus, techniques able to accurately monitor the real-time evolution of ablation and the temperature change of internal structures are needed. The aim is to check that the tumor region is completely covered by the ablation area and that the healthy tissue is preserved [90,91]. EIT is considered a suitable technique for this purpose, as temperature elevation impacts tissue conductivity through the heated domain, with centimeter-scale spatial resolution [90,91]. In addition, an enhancement in the sensitivity occurs if the RFA probe is exploited as a further electrode, which leads to a partially invasive version of EIT [90].

EIT has been studied to monitor both the changes in temperature during cooling and during and after heating [91]. The studies were conducted on phantoms and on realistic human simulations [90,91], but in vivo experiments still need to be conducted. It is also important to critically use a priori information, for example, information coming from other diagnostic techniques such as MRIs or form literature surveys, in order to obtain accurate estimates of the temperature in the living tissues that are going to be treated [90]. In general, EIT was able to monitor the complete ablation of a tissue target, but prevention of undesired overheating and damage to healthy tissue is still under study [90]. In addition, in order to improve resolution and accuracy, future applications should be focused on the impact of movement, electronic noise and carbonization above certain temperatures [90,91].

### 3.4. Tumor Detection

Because many tumors are characterized by significantly different conductivity and permittivity from surrounding normal tissues, EIT might be able to identify them by exploiting these different electrical properties [92,93,94]. Indeed, an increasing number of studies are investigating the use of EIT for screening and early detection of cancer, with breast cancer being the main application in addition to skin, thyroid, liver, cervix and lung cancer [92,93,95,96,97,98,99,100,101,102]. In breast imaging, 3D maps of conductivity distributions have reported detectable breast cancer models sized about 12–14 mm [103], up to 5 mm with 4.9 mΩ sensitivity [104] with 3D systems of respectively 128 and 90 electrodes. Because EIT operates generally at frequencies lower than 1 MHz, while the relevant information for tumor detection can be derived from the tumor impedance spectra, which employ a higher-frequency domain, the CCERT (capacitively coupled electrical resistance tomography) technique is more suitable than traditional EIT for cancer detection [95]. Although EIT is a very good technique for dynamical imaging, in the context of tumor diagnosis, time-difference imaging is less plausible, as it is unrealistic to obtain the reference data of the patient before the development of the tumor (i.e., baseline) [95]. Despite this, EIT techniques have been shown to be able to successfully localize and discriminate lesions and pathological sites of cancer from normal tissues in both phantom-based simulations and in vivo applications [92,93,95,96,97,98,99,100,101,102].

### 3.5. Assessment of Muscle Health

EIT could substitute electrical impedance myography, which is a non-invasive and painless method used to assess muscle health [105]. EIT could indeed overcome some of the main limitations shown by this technique, such as its dependence on skin/adipose-tissue thickness and its inability to distinguish closely spaced muscle groups and to assess the heterogeneity of muscle tissue. Conducted experiments have also shown that the coupling of an ultrasound (US) device and an EIT system could provide the best and most complete information, with improvements in the image accuracy. Studies conducted on phantoms, simulation systems and patients have shown the coupled US/EIT system to be successful in capturing different physiological and pathological muscle characteristics by detecting changes in the electrical properties of muscles. Specifically, the capability of US/EIT systems to discriminate pathological from physiological conditions by detecting differences in muscle (longitudinal and transverse) conductivity and permittivity suggest that EIT is a promising tool for non-invasive and spatially localized assessment of muscle health [105].

## 4. Wearable Solutions

### 4.1. Hardware Characteristics

A wearable device [106] is a system that can be worn on the human body or embedded in clothing. It mainly consists of receptors, such as electrodes or microminiaturized sensors, capable of detecting and sending the acquired data, through standard communication protocols, to a processing unit. Thanks to dedicated software and algorithms, the collected signals can be extrapolated to extract useful information on a PC or smartphone. Most of the portable EIT systems are intended to be utilized both in clinic and at home for daily non-invasive monitoring; they usually involve a belt, or a wrist wrap with embedded electrodes. Portable EIT systems are designed to follow physiological changes in the body through changes in pulmonary gas and body fluid.

At first, researchers focused their attention mainly on the microminiaturization of the conventional EIT system [107], trying to solve the issue of bulky volume, and on the wireless transmission of data. Additionally, their module can pre-filter the acquired signals and perform the digital processing on a separated FPGA.

#### 4.1.1. Low Power Consumption

For portable applications, it is necessary to achieve a low power consumption because the system is powered through portable energy sources, like batteries, that have limited power availability and tend to deteriorate with time; it is then necessary to achieve a high output impedance in the current driver stage to maximize the current flowing in the load [56]. The current generator could be oscillator-based or DAC-based; the latter is preferred in portable devices because it has lower power consumption [49]. For what concerns the ADC, to reduce power consumption in the ADC SAR and avoid oversampling, lower frequencies can be processed at a lower sampling rate [61]. Some specific modifications have also been implemented in the demodulation stage. In [46,104], I-Q demodulation is proposed; this method is effective in terms of power consumption because the subsequent steps of the signal processing chain are operated at lower frequencies and no additional low pass filters are required, but the input referred noise is higher. In the cited works, fast I-Q demodulation is performed to reduce power consumption: the settling time is reduced for signals at 10 kHz to allow a 5 frames/s real-time operation.

#### 4.1.2. Electrode Configuration

As reported above in Section 2.3.6, in the design of a wearable device, particular attention must be paid to the choice of the electrodes. The main sources of limitation are the intra- and inter-operator variability in the applications of the electrodes, which require feasibility for both clinical and domestic settings, for long-term usage which causes the drying of the conductive gel needed for Ag/AgCl electrodes and mainly for usage during motion which creates artifacts. The most used solution is the application of a belt of dry electrodes made of a conductive fabric. Experiments have been carried out, and it has been proved that there are no differences compared to Ag/AgCl electrode measurements [48]. However, dry electrodes can be more easily displaced, and this might lead to motion artifacts. An interesting solution is the use of a wearable wireless belt with dry electrodes combined with the Gauss–Newton method for the optimization of the EIT image [36]. This study emphasizes that a lower risk of skin irritation implies better durability and increased comfort obtained with dry electrodes. Moreover, results demonstrated accurate location information of the objects in the images thanks to the reconstruction algorithm and that, in a human experiment, the difference between the ratios of lung area to the whole chest when inhaling and exhaling fit the physiological changes of normal lung activity. Studies based on the use of textile electrodes integrated with a clothing belt have been reported, as previously mentioned.

In addition to the basic requirements for the voltage acquisition circuits in traditional EIT, wearable devices may require more stringent characteristics in terms of noise and the input dynamic range of the electrodes due to smaller amplitudes of the signals [49].

#### 4.1.3. Examples of Wearable EIT Implementations

In this work we report as examples two representative hardware solutions presented in studies conducted for human lung ventilation. The first study [48] focuses on a belt which includes 16 embossed nanofiber web electrodes, designed to make good contact with the skin, made of Ag-plated PVDF nanofiber web and metallic threads. Thanks to this, it reaches improved comfort and a reduced contact impedance thanks to a large contact area and padding behind each electrode; contact impedance and stability were found to be comparable to those of Ag/AgCl electrodes. The designed belt includes an inner band that can be stretched, allowing it to fit a range of thorax sizes. Moreover, to better adapt the shape to the chest, sponges have been placed in pockets on the outer band to allow tight contact with the body. Finally, a standard ECG electrode provides a reference for the EIT acquisition system. Even if the proposed electrodes allow larger resting noise levels, the performance of the belt-mounted electrodes over time has been found to be more stable than that of adhesive ones.

The second work [47] concerns a belt including 16 textile electrodes, made of cotton inside with silver wire cloth on the surface. Experimental results, consistent with the ones from the commercial ECG electrodes, proved the validity of the proposed device, and the impedance fields were found to be larger, thus providing better imaging discrimination.

Other proposed solutions are capable of acquiring signals simultaneously, and an example is provided by a system based on a novel solution of cooperative sensors [108]. The work presents a sensing architecture for frequency-multiplexed EIT and synchronous ECG data acquisition. The advantages of this structure are a significant reduction in the cabling complexity and flexible EIT stimulation and measurement patterns. Cooperative sensors use a bidirectional communication bus to digitally transmit the information between the master device and the sensors, which only have contact with the skin. In the proposed system the master device is placed above the belt on the right ventral part of the thorax, while the 16 sensors are distributed equidistantly on the transverse plane, connected to the elastic belt; additionally, 4 sensors are placed in the infraclavicular regions for ECG data acquisition. The main advantage is that the device provides continuous measurements, as the sensors automatically turn on when they come into contact with the skin until the patient takes off the vest. Data are stored in the master device and transmitted to a computer via wi-fi at the end of the acquisition session.

### 4.2. Applications of Wearable EIT

Wearable EIT devices have been proposed in various fields, among which are pulmonary imaging, cancer detection and gesture recognition. Furthermore, such devices have been previously integrated with other measurement systems to obtain multiparametric monitoring.

#### 4.2.1. Pulmonary Imaging

Pulmonary imaging is the most prominent application for wearable devices. Monitoring lung resistivity has been recently proposed [109] for the detection of pulmonary edema, as the change in the proportion of liquid and air leads to different electrical properties between edematous and healthy lung tissue. These devices combine the transthoracic and EIT approaches to obtain a low-cost, continuous over long-term periods and safe system, without the need for ionizing radiation, with the aim of obtaining the left and right lung resistivity values and the ECG signal, valuable for the synchronization of the procedure. Unfortunately, there are still some disadvantages, as the former approach cannot directly measure the impedance of internal organs, and it is largely dependent on anthropometric parameters, while the latter needs many electrodes and is highly sensitive to measurement noise. Another study designed for cardio-pulmonary monitoring [42] proposed a system with 32 active electrodes, each of them provided with an application-specific integrated circuit (ASIC) mounted on a flexible printed circuit board wrapped inside the belt. The innovative feature of this device is the employment of two parallel EIT data acquisition channels to achieve a frame rate of 107 fps, which makes it one of fastest wearable systems. The system is also capable of recording other signals, including heart rate, environmental temperature and humidity, and tracking the thorax shape and laying position to aid in EIT model selection.

#### 4.2.2. Cancer Detection

A high-resolution EIT integrated circuit has been proposed for early breast cancer detection. Researchers have assembled a portable EIT system, able to detect a 5 mm cancer mass, into a brassier shape [29]. The circuit has been integrated via a multi-layered fabric circuit board which includes 90 electrodes arranged in five concentric circles and 2 reference electrodes for current stimulation and voltage sensing. Researchers have conducted simulations to determine the minimum number of sensors required for a good sensitivity, which turned out to be 80. The final device is compact, highly sensitive and can be connected to a mobile smart device for early breast cancer detection.

#### 4.2.3. Gesture Recognition

A widely studied application is gesture recognition, in which the EIT device is used to measure the inner conductivity distributions caused by bone and muscle movement of the forearm. An example has been proposed in [110], which presents a wearable device composed of a wrist wrap with embedded electrodes, which passes the data to a deep learning neural network for gesture recognition; the system is able to recognize 19 hand gestures, with an accuracy of 98%, with the round-robin sub-grouping method. To optimize the system, the number of robins could be reduced while maintaining high classification accuracy. Another example has been published recently [111] and is based on the two-terminal EIT technique and machine learning algorithms. The device can recognize nine different gestures, and it works with a speed of 8 frames per second. Compared with previous works, it requires fewer electrodes, and it gains about 98.5% in accuracy, thanks to the quadratic discriminant algorithm it uses as a classification model.

To maintain data quality, these EIT devices should not be used with other systems measuring bioimpedance, as the injected high frequency currents may influence the signals. Future work will entail optimizing the proposed systems by improving the spatial resolution. Moreover, further tests must be conducted to investigate the feasibility of monitoring portable devices on humans.

#### 4.2.4. Multi-Parameter EIT

Finally, wearable EIT solutions have focused on designing a belt for thorax vital multiple sign monitoring. A system with 16 active electrodes, each connected to a specific integrated circuit (ASIC), has been proposed [44]. This architecture allows programmable, flexible electrode current drive and voltage-sensing patterns under simple digital control. The device can capture high-quality lung ventilation images with a frame rate of 122 fps, as well as breathing cycle, heart rate and boundary shape information. The innovative feature introduced in this work is the high-performance ASIC that provides a superior common-mode rejection ratio while reducing the complexity of the wiring and digital control. A similar solution was introduced a few years earlier [43] and comprises an active electrode integrated circuit made of a wideband high-power current driver, a low noise voltage amplifier and two shape sensor buffers.

The diagnosis of sleep apnea and hypoventilation to supplement polysomnography and home sleep tests have also been investigated, with devices capable of obtaining a continuous tidal volume signal from real-time EIT lung ventilation images [112].

### 4.3. Integration in Telemedicine Platforms

As was mentioned before, some wearable EIT devices already have associated mobile applications that allow access to the results in a portable fashion. In general, wearable EIT can be integrated into body area networks (BANs) to become part of telemedicine platforms. Specifically, BANs or body sensor networks (BSNs) are systems composed of a network of wearable devices that can be implanted in the body, placed on the body in fixed positions or carried by the person in clothes’ pockets, by hand or in a bag [113]. Wearable EIT devices fall in the second category, i.e., they are placed on the body in fixed positions.

Multiple examples have been mentioned that include other sensors in addition to the wearable EIT device. In the section dedicated to wearable EIT, an ECG device was often cited as part of the system, due to the ease of positioning in a belt. Some devices [46] have been equipped with a sensor system that provides more types of signals such as electrical biopotentials like ECG and EMG, bio-impedance and moreover temperature and humidity signals. These devices include a belt embedded with 16 electrodes (current and potential electrodes) which measure both ECG and EIT signals. Other proposed solutions [108] are able to acquire signals simultaneously from a wearable EIT device, an ECG, a three-axial accelerometer to measure body movement and a pulse oximeter to estimate peripheral blood oxygen saturation. For the continuous measuring of multi-channel signals to estimate continuous tidal volume [112], a wearable system proposed in the study includes a chest belt (16 electrodes for EIT imaging and ECG data acquisition), a nasal canula and nasal pressure sensor, a finger sensor, a microphone to record snoring sound, an accelerometer, a gyroscope and a magnetometer to detect body position; the multi-parameter module containing the sensors and circuits needed is in the middle of the chest and is controlled by an FPGA.

**Figure 3:**
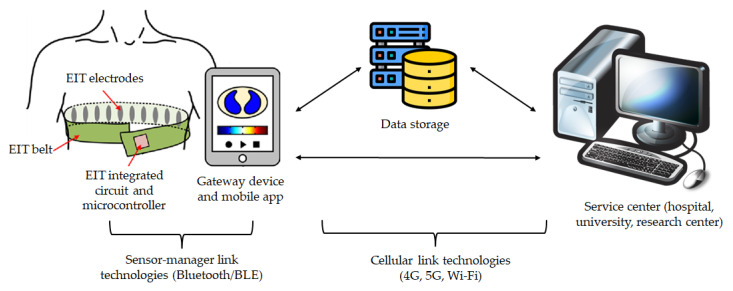
Telemedicine system with EIT device following the two-hop architecture; adapted from [115,116]. The EIT electrodes are connected to the microcontroller and other components; all these elements are placed on a belt. Data are sent via sensor-manager link technologies, such as Bluetooth, to a gateway device, generally a smartphone or tablet. A mobile app can be installed on the gateway device for real-time image visualization. The gateway device sends data via cellular link technologies to a central database (data storage), and results can be accessed from a remote service center.

Telemedicine platforms generally follow a two-hop architecture; an example with an EIT device is shown in Figure 3 [114]. Data coming from sensors are transmitted to a gateway with sensor-manager link technologies and then from the gateway to the data management section with cellular link technologies.

In other cases, like one of the studies mentioned before [108], there is no sensor-manager link technology because the signal-acquiring devices are connected with a cellular-link technology like wi-fi to the internet. This is often the case when sensor-containing garments [115] are used, as sensors can be connected to a master data logger with cables. Wearable EIT devices can be considered smart garments and may provide more signals of interest simultaneously, as was highlighted in the previous section.

## 5. Discussion

In this review we have focused on the development of EIT hardware design and applications, from classical to wearable solutions. EIT has demonstrated peculiar features as a medical imaging modality. It is non-invasive, does not require ionizing radiation and is characterized by high temporal resolution. Moreover, the low-cost equipment and the good portability make it suitable both for long-term and real-time bedside monitoring, for instance in the case of patients that cannot be easily transported to other parts of a hospital, such as ICU patients. Major limitations include the lower spatial resolution compared to other imaging techniques, the low sensitivity to phenomena of interest and the high sensitivity to any imperfections in the hardware and the electrode–body contact. Nevertheless, together with the increased scientific interest in EIT, the number of studies aiming at optimizing measurement configurations and improving reconstruction algorithms has increased [117]. Moreover, the COVID-19 pandemic has represented the perfect scenario for the application of EIT, providing a further boost to its technological development and application.

As discussed in detail in Section 4, EIT systems have tended towards wearable solutions, with increased portability, lower power consumption and application-specific design. The wearable approach permits the use of a wireless EIT module of small volume, thus reducing the encumbrance of EIT systems [107]. In wearable systems the EIT electrodes can be embedded into clothes and used under motion with good results thanks to some adaptations.

The possibility of having wearable and wireless EIT devices allows the introduction of these systems into more scenarios, such as remote monitoring of patients, even during their daily life. Wearable EIT devices can also be used as complements to other diagnostic techniques, which can be requested in the case of anomalies detected by the continuous monitoring of EIT, i.e., using EIT technology as an “early-warning system”.

## 6. Conclusions

EIT is a medical imaging technique based on the injection of a current or voltage pattern through electrodes on the skin of the patient and on the reconstruction of the internal conductivity distribution from the voltages collected by the electrodes. Compared to other imaging techniques, EIT has relevant advantages. In fact, EIT does not use ionizing radiation, is non-invasive and is characterized by high temporal resolution. Moreover, its low cost and high portability make it suitable for real-time, bedside monitoring. However, EIT is also characterized by poor spatial resolution, and for this reason it cannot be used in place of other medical imaging techniques, but rather as an additional instrument whenever the other techniques cannot be used.

The possibility of designing wearable devices based on EIT has recently given a boost to this technology. In this paper the current literature on EIT systems has been reviewed, from the hardware design to its clinical applications. The development of wireless and wearable EIT devices might reinforce the method’s use in the clinical setting and broaden its field of application to new situations, such as remote monitoring in non-controlled environments like patients’ homes.

## Figures and Tables

**Table 1:** Advantages and disadvantages of EIT imaging modalities.

Imaging Modality	Output	Application	Advantages (+) and Disadvantages (−)
Absolute	Absolute impedance	Computer simulations	Always applicable (+) Sensitive to instrumentation and modelling errors (−) Not reliable in a clinical setting (−)
Time- difference	Time-dependent impedance change	Time-varying phenomena	Robust to modelling errors (+) Time-referenced data required (−)
Frequency-difference	Frequency-dependent impedance change	Identification of different tissues	Robust to modelling errors (+) Time-referenced data not required (+) Sufficient contact between tissues’ electrical frequency properties required (−)

**Table 2:** Issues, challenges and innovative solutions found in the choice and design of electrodes.

Issue	Challenge	Innovative Solutions
Optimal number	Trade-off between image resolution and computation time	Rotational EIT
Correct positioning	Lack of robustness of image reconstruction algorithms	Elastic or adjustable electrode belt
Contact impedance	Modelling errors	Multi-pole measurement strategy; simultaneous reconstruction of electrodes and electrical properties; capacitively coupled EIT
Noise at the interface	Degradation of image resolution	Active electrode-based EIT
Ensure good contact	Variable contact impedance and reconstruction errors	Conductive gel; faulty contact detection algorithm
Ensure good contact (wearable systems)	Variable contact impedance and reconstruction errors	Dry and flexible electrodes

## Data Availability

Not applicable.

## References

[1] Bayford R., Tizzard A. (2012). Bioimpedance Imaging: An Overview of Potential Clinical Applications. Analyst.

[2] Rafiei-Naeini M., McCann H. (2008). Low-Noise Current Excitation Sub-System for Medical EIT. Physiol. Meas..

[3] Sadleir R.J., Fox R.A., Turner V.F. (2000). Inflatable belt for the application of electrode arrays. Rev. Sci. Instrum..

[4] Dong G., Bayford R., Liu H., Zhou Y., Yan W. EIT Images with Improved Spatial Resolution Using a Realistic Head Model. Proceedings of the Annual International Conference of the IEEE Engineering in Medicine and Biology Society.

[5] Liu S., Jia J., Zhang Y.D., Yang Y. (2018). Image Reconstruction in Electrical Impedance Tomography Based on Structure-Aware Sparse Bayesian Learning. IEEE Trans. Med. Imaging.

[6] Brown B.H. (2003). Electrical Impedance Tomography (EIT): A Review. J. Med. Eng. Technol..

[7] Lionheart W.R.B. (2004). EIT Reconstruction Algorithms: Pitfalls, Challenges and Recent Developments. Physiol. Meas..

[8] Dijkstra A.M., Brown B.H., Leathard A.D., Harris N.D., Barber D.C., Edbrooke D.L. (1993). Review Clinical Applications of Electrical Impedance Tomography. J. Med. Eng. Technol..

[9] Khalil S.F., Mohktar M.S., Ibrahim F. (2014). The Theory and Fundamentals of Bioimpedance Analysis in Clinical Status Monitoring and Diagnosis of Diseases. Sensors.

[10] Gabriel S., Lau R.W., Gabriel C. (1996). The Dielectric Properties of Biological Tissues: III. Parametric Models for the Dielectric Spectrum of Tissues. Phys. Med. Biol..

[11] Nopp P., Rapp E., Pfutzner H., Nakesch H., Rusham C. (1993). Dielectric Properties of Lung Tissue as a Function of Air Content. Phys. Med. Biol..

[12] Schwan H.P. (1957). Electrical Properties of Tissue and Cell Suspensions. Adv. Biol. Med. Phys..

[13] Gabriel C., Gabriel S., Corthout E. (1996). The Dielectric Properties of Biological Tissues: I. Literature Survey. Phys. Med. Biol..

[14] Kyle U.G., Bosaeus I., De Lorenzo A.D., Deurenberg P., Elia M., Gómez J.M., Heitmann B.L., Kent-Smith L., Melchior J.C., Pirlich M. (2004). Bioelectrical Impedance Analysis—Part I: Review of Principles and Methods. Clin. Nutr..

[15] Packham B., Koo H., Romsauerova A., Ahn S., McEwan A., Jun S.C., Holder D.S. (2012). Comparison of Frequency Difference Reconstruction Algorithms for the Detection of Acute Stroke Using EIT in a Realistic Head-Shaped Tank. Physiol. Meas..

[16] Lee J.M., Uhlmann G. (1989). Determining Anisotropic Real-analytic Conductivities by Boundary Measurements. Commun. Pure Appl. Math..

[17] Khan T.A., Ling S.H. (2019). Review on Electrical Impedance Tomography: Artificial Intelligence Methods and Its Applications. Algorithms.

[18] Brazey B., Haddab Y., Zemiti N. (2022). Robust Imaging Using Electrical Impedance Tomography: Review of Current Tools. Proc. R. Soc. A Math. Phys. Eng. Sci..

[19] Riera J., Riu P.J., Casan P., Masclans J.R. (2011). Tomografía de Impedancia Eléctrica En La Lesión Pulmonar Aguda. Med. Intensiv..

[20] Nissinen A., Kolehmainen V.P., Kaipio J.P. (2011). Compensation of Modelling Errors Due to Unknown Domain Boundary in Electrical Impedance Tomography. IEEE Trans. Med. Imaging.

[21] Adler A., Holder D. (2021). Electrical Impedance Tomography: Methods, History and Applications.

[22] Seo J.K., Harrach B., Woo E.J. (2009). Recent Progress on Frequency Difference Electrical Impedance Tomography. ESAIM Proc..

[23] Wu C., Soleimani M. (2019). Frequency Difference EIT with Localization: A Potential Medical Imaging Tool during Cancer Treatment. IEEE Access.

[24] Takhti M., Odame K. (2019). Structured Design Methodology to Achieve a High SNR Electrical Impedance Tomography. IEEE Trans. Biomed. Circuits Syst..

[25] Anand S., Jandial P., Nersisson R. A Technical Survey on Hardware Configurations for Electrical Impedance Tomography Systems. Proceedings of the 3rd IEEE International Virtual Conference on Innovations in Power and Advanced Computing Technologies, i-PACT 2021.

[26] Harikumar R., Prabu R., Raghavan S. (2013). Electrical Impedance Tomography (EIT) and Its Medical Applications: A Review. Int. J. Soft Comput. Eng..

[27] Rosa B.M.G., Yang G.Z. (2020). Bladder Volume Monitoring Using Electrical Impedance Tomography with Simultaneous Multi-Tone Tissue Stimulation and DFT-Based Impedance Calculation Inside an FPGA. IEEE Trans. Biomed. Circuits Syst..

[28] Halter R.J., Hartov A., Paulsen K.D. (2008). A Broadband High-Frequency Electrical Impedance Tomography System for Breast Imaging. IEEE Trans. Biomed. Eng..

[29] Hong S., Lee K., Ha U., Kim H., Lee Y., Kim Y., Yoo H.J. (2015). A 4.9 MΩ-Sensitivity Mobile Electrical Impedance Tomography IC for Early Breast-Cancer Detection System. IEEE J. Solid-State Circuits.

[30] Kim M., Jang J., Kim H., Lee J., Lee J., Lee J., Lee K.R., Kim K., Lee Y., Lee K.J. (2017). A 1.4-m Ω-Sensitivity 94-DB Dynamic-Range Electrical Impedance Tomography SoC and 48-Channel Hub-SoC for 3-D Lung Ventilation Monitoring System. IEEE J. Solid-State Circuits.

[31] Xu G., Wang R., Zhang S., Yang S., Justin G.A., Sun M., Yan W. A 128-Electrode Three Dimensional Electrical Impedance Tomography System. Proceedings of the Annual International Conference of the IEEE Engineering in Medicine and Biology.

[32] Huang C.N., Yu F.M., Chung H.Y. (2007). Rotational Electrical Impedance Tomography. Meas. Sci. Technol..

[33] Lehti-Polojarvi M., Koskela O., Seppanen A., Figueiras E., Hyttinen J. (2018). Rotational Electrical Impedance Tomography Using Electrodes with Limited Surface Coverage Provides Window for Multimodal Sensing. Meas. Sci. Technol..

[34] Murphy E.K., Mahara A., Halter R.J. (2017). Absolute Reconstructions Using Rotational Electrical Impedance Tomography for Breast Cancer Imaging. IEEE Trans. Med. Imaging.

[35] Gaggero P.O., Adler A., Brunner J., Seitz P. (2012). Electrical Impedance Tomography System Based on Active Electrodes. Physiol. Meas..

[36] Lin B.S., Yu H.R., Kuo Y.T., Liu Y.W., Chen H.Y., Lin B.S. (2022). Wearable Electrical Impedance Tomography Belt With Dry Electrodes. IEEE Trans. Biomed. Eng..

[37] Boone K.G., Holder D.S. (1996). Effect of Skin Impedance on Image Quality and Variability in Electrical Impedance Tomography: A Model Study. Med. Biol. Eng. Comput..

[38] Hua P., Woo E.J., Webster J.G., Tompkins W.J. (1993). Using Compound Electrodes In Electrical Impedance Tomography. IEEE Trans. Biomed. Eng..

[39] Agnelli J.P., Kolehmainen V., Lassas M.J., Ola P., Siltanen S. (2021). Simultaneous Reconstruction of Conductivity, Boundary Shape, and Contact Impedances in Electrical Impedance Tomography. SIAM J. Imaging Sci..

[40] Jiang Y.D., Soleimani M. (2019). Capacitively Coupled Electrical Impedance Tomography for Brain Imaging. IEEE Trans. Med. Imaging.

[41] Wu Y., Hanzaee F.F., Jiang D., Bayford R.H., Demosthenous A. (2021). Electrical Impedance Tomography for Biomedical Applications: Circuits and Systems Review. IEEE Open J. Circuits Syst..

[42] Wu Y., Jiang D., Bardill A., De Gelidi S., Bayford R., Demosthenous A. (2018). A High Frame Rate Wearable EIT System Using Active Electrode ASICs for Lung Respiration and Heart Rate Monitoring. IEEE Trans. Circuits Syst. I Regul. Pap..

[43] Wu Y., Langlois P., Bayford R., Demosthenous A. Design of a CMOS Active Electrode IC for Wearable Electrical Impedance Tomography Systems. Proceedings of the IEEE International Symposium on Circuits and Systems.

[44] Wu Y., Jiang D., Bardill A., Bayford R., Demosthenous A. (2019). A 122 Fps, 1 MHz Bandwidth Multi-Frequency Wearable EIT Belt Featuring Novel Active Electrode Architecture for Neonatal Thorax Vital Sign Monitoring. IEEE Trans. Biomed. Circuits Syst..

[45] Asfaw Y., Adler A. (2005). Automatic Detection of Detached and Erroneous Electrodes in Electrical Impedance Tomography. Physiol. Meas..

[46] Rymarczyk T., Nita P., Vejar A., Wos M., Oleszek M., Adamkiewicz P. Architecture of a Mobile System for the Analysis of Biomedical Signals Based on Electrical Tomography. Proceedings of the 2018 Applications of Electromagnetics in Modern Techniques and Medicine, PTZE 2018.

[47] Hu C.L., Cheng I.C., Huang C.H., Liao Y.T., Lin W.C., Tsai K.J., Chi C.H., Chen C.W., Wu C.H., Lin I.T. (2021). Dry Wearable Textile Electrodes for Portable Electrical Impedance Tomography. Sensors.

[48] Oh T.I., Kim T.E., Yoon S., Kim K.J., Woo E.J., Sadleir R.J. (2012). Flexible Electrode Belt for EIT Using Nanofiber Web Dry Electrodes. Physiol. Meas..

[49] Van Helleputte N., Konijnenburg M., Pettine J., Jee D.W., Kim H., Morgado A., Van Wegberg R., Torfs T., Mohan R., Breeschoten A. (2015). A 345 Μw Multi-Sensor Biomedical SoC with Bio-Impedance, 3-Channel ECG, Motion Artifact Reduction, and Integrated DSP. IEEE J. Solid-State Circuits.

[50] (2015). Medical Electrical Equipment Part 1: General Requirements for Basic Safety and Essential Performance.

[51] Zeng L., Heng C.H. (2021). An 8-Channel 1.76-MW 4.84-Mm2Electrical Impedance Tomography SoC with Direct If Frequency Division Multiplexing. IEEE Trans. Circuits Syst. II Express Briefs.

[52] Wu Y., Jiang D., Langlois P., Bayford R., Demosthenous A. A CMOS Current Driver with Built-in Common-Mode Signal Reduction Capability for EIT. Proceedings of the ESSCIRC 201743rd IEEE European Solid State Circuits Conference.

[53] Langlois P.J., Wu Y., Bayford R.H., Demosthenous A. (2015). On the Application of Frequency Selective Common Mode Feedback for Multifrequency EIT. Physiol. Meas..

[54] Wicaksono R., Baidillah M.R., Darma P.N., Inoue A., Tsuji H., Takei M. (2021). Pocket Electrical Impedance Tomography (p-EIT) System with Wide Impedance Range Buffer- Mirrored Current Source (BMCS) with Assist of Filter-Trained Quasi-3-D Method for Functional Gastric-Shape Imaging. IEEE Trans. Instrum. Meas..

[55] Xu Y., Yan Z., Han B., Dong F. (2021). An FPGA-Based Multifrequency EIT System with Reference Signal Measurement. IEEE Trans. Instrum. Meas..

[56] Li W., Xia J., Zhang G., Ma H., Liu B., Yang L., Zhou Y., Dong X., Fu F., Shi X. (2019). Fast High-Precision Electrical Impedance Tomography System for Real-Time Perfusion Imaging. IEEE Access.

[57] Shahghasemi M., Odame K.M. A Wide-Band, Wide-Swing Current Driver for Electrical Impedance Tomography Applications. Proceedings of the Midwest Symposium on Circuits and Systems.

[58] Shi X., Li W., You F., Huo X., Xu C., Ji Z., Liu R., Liu B., Li Y., Fu F. (2018). High-Precision Electrical Impedance Tomography Data Acquisition System for Brain Imaging. IEEE Sens. J..

[59] Shi X., You F., Xu C., Ji Z., Liu R., Dong X., Fu F., Huo X. Design and Implementation of a High-Precision Electrical Impedance Tomography Data Acquisition System for Brain Imaging. Proceedings of the 2016 IEEE Biomedical Circuits and Systems Conference, BioCAS 2016.

[60] Yang D., Huang G., Xu B., Wang X., Wang Z., Wei Z. (2022). A DSP-Based EIT System with Adaptive Boundary Voltage Acquisition. IEEE Sens. J..

[61] Takhti M., Teng Y.C., Odame K. (2018). A 10 MHz Read-Out Chain for Electrical Impedance Tomography. IEEE Trans. Biomed. Circuits Syst..

[62] Tomicic V., Cornejo R. (2019). Lung Monitoring with Electrical Impedance Tomography: Technical Considerations and Clinical Applications. J. Thorac. Dis..

[63] Visser K.R. (1989). Electric Properties of Flowing Blood and Impedance Cardiography. Ann. Biomed. Eng..

[64] Wrigge H., Zinserling J., Muders T., Varelmann D., Günther U., Von Der Groeben C., Magnusson A., Hedenstierna G., Putensen C. (2008). Electrical Impedance Tomography Compared with Thoracic Computed Tomography during a Slow Inflation Maneuver in Experimental Models of Lung Injury. Crit. Care Med..

[65] Frerichs I., Hinz J., Herrmann P., Weisser G., Hahn G., Dudykevych T., Quintel M., Hellige G. (2002). Detection of Local Lung Air Content by Electrical Impedance Tomography Compared with Electron Beam CT. J. Appl. Physiol..

[66] Richard J.C., Pouzot C., Gros A., Tourevieille C., Lebars D., Lavenne F., Frerichs I., Guérin C. (2009). Electrical Impedance Tomography Compared to Positron Emission Tomography for the Measurement of Regional Lung Ventilation: An Experimental Study. Crit. Care.

[67] Hinz J., Moerer O., Neumann P., Dudykevych T., Hellige G., Quintel M. (2005). Effect of Positive End-Expiratory-Pressure on Regional Ventilation in Patients with Acute Lung Injury Evaluated by Electrical Impedance Tomography. Eur. J. Anaesthesiol..

[68] Coulombe N., Gagnon H., Marquis F., Skrobik Y., Guardo R. (2005). A Parametric Model of the Relationship between EIT and Total Lung Volume. Physiol. Meas..

[69] Meier T., Luepschen H., Karsten J., Leibecke T., Großherr M., Gehring H., Leonhardt S. (2008). Assessment of Regional Lung Recruitment and Derecruitment during a PEEP Trial Based on Electrical Impedance Tomography. Intensive Care Med..

[70] Zick G., Elke G., Becher T., Schädler D., Pulletz S., Freitag-Wolf S., Weiler N., Frerichs I. (2013). Effect of PEEP and Tidal Volume on Ventilation Distribution and End-Expiratory Lung Volume: A Prospective Experimental Animal and Pilot Clinical Study. PLoS ONE.

[71] Odenstedt H., Lindgren S., Olegård C., Erlandsson K., Lethvall S., Åneman A., Stenqvist O., Lundin S. (2005). Slow Moderate Pressure Recruitment Maneuver Minimizes Negative Circulatory and Lung Mechanic Side Effects: Evaluation of Recruitment Maneuvers Using Electric Impedance Tomography. Intensive Care Med..

[72] Van Heerde M., Roubik K., Kopelent V., Kneyber M.C.J., Markhorst D.G. (2010). Spontaneous Breathing during High-Frequency Oscillatory Ventilation Improves Regional Lung Characteristics in Experimental Lung Injury. Acta Anaesthesiol. Scand..

[73] Costa E.L.V., Chaves C.N., Gomes S., Beraldo M.A., Volpe M.S., Tucci M.R., Schettino I.A.L., Bohm S.H., Carvalho C.R.R., Tanaka H. (2008). Real-Time Detection of Pneumothorax Using Electrical Impedance Tomography. Crit. Care Med..

[74] Frerichs I., Schiffmann H., Hahn G., Hellige G. (2001). Non-Invasive Radiation-Free Monitoring of Regional Lung Ventilation in Critically Ill Infants. Intensive Care Med..

[75] Miedema M., De Jongh F.H., Frerichs I., Van Veenendaal M.B., Van Kaam A.H. (2011). Changes in Lung Volume and Ventilation during Surfactant Treatment in Ventilated Preterm Infants. Am. J. Respir. Crit. Care Med..

[76] Van Veenendaal M.B., Miedema M., De Jongh F.H.C., Van Der Lee J.H., Frerichs I., Van Kaam A.H. (2009). Effect of Closed Endotracheal Suction in High-Frequency Ventilated Premature Infants Measured with Electrical Impedance Tomography. Intensive Care Med..

[77] Deibele J.M., Luepschen H., Leonhardt S. (2008). Dynamic Separation of Pulmonary and Cardiac Changes in Electrical Impedance Tomography. Physiol. Meas..

[78] Frerichs I., Hinz J., Herrmann P., Weisser G., Hahn G., Quintel M., Hellige G. (2002). Regional Lung Perfusion as Determined by Electrical Impedance Tomography in Comparison with Electron Beam CT Imaging. IEEE Trans. Med. Imaging.

[79] Shono A., Kotani T., Frerichs I. (2021). Personalisation of Therapies in COVID-19 Associated Acute Respiratory Distress Syndrome, Using Electrical Impedance Tomography. J. Crit. Care Med..

[80] Mauri T., Spinelli E., Scotti E., Colussi G., Basile M.C., Crotti S., Tubiolo D., Tagliabue P., Zanella A., Grasselli G. (2020). Potential for Lung Recruitment and Ventilation-Perfusion Mismatch in Patients with the Acute Respiratory Distress Syndrome from Coronavirus Disease 2019. Crit. Care Med..

[81] Pulletz S., Van Genderingen H.R., Schmitz G., Zick G., Schädler D., Scholz J., Weiler N., Frerichs I. (2006). Comparison of Different Methods to Define Regions of Interest for Evaluation of Regional Lung Ventilation by EIT. Physiol. Meas..

[82] Borgmann S., Linz K., Braun C., Dzierzawski P., Spassov S., Wenzel C., Schumann S. (2022). Lung Area Estimation Using Functional Tidal Electrical Impedance Variation Images and Active Contouring. Physiol. Meas..

[83] Ke X.Y., Hou W., Huang Q., Hou X., Bao X.Y., Kong W.X., Li C.X., Qiu Y.Q., Hu S.Y., Dong L.H. (2022). Advances in Electrical Impedance Tomography-Based Brain Imaging. Mil. Med. Res..

[84] Hannan S., Faulkner M., Aristovich K., Avery J., Walker M., Holder D. (2018). Imaging Fast Electrical Activity in the Brain during Ictal Epileptiform Discharges with Electrical Impedance Tomography. NeuroImage Clin..

[85] Hannan S., Faulkner M., Aristovich K., Avery J., Walker M.C., Holder D.S. (2020). In Vivo Imaging of Deep Neural Activity from the Cortical Surface during Hippocampal Epileptiform Events in the Rat Brain Using Electrical Impedance Tomography. Neuroimage.

[86] Holder D.S. (1992). Detection of Cortical Spreading Depression in the Anaesthetised Rat by Impedance Measurement with Scalp Electrodes: Implications for Non-Invasive Imaging of the Brain with Electrical Impedance Tomography. Clin. Phys. Physiol. Meas..

[87] Boverman G., Kao T.J., Wang X., Ashe J.M., Davenport D.M., Amm B.C. (2016). Detection of Small Bleeds in the Brain with Electrical Impedance Tomography. Physiol. Meas..

[88] Fu F., Li B., Dai M., Hu S.J., Li X., Xu C.H., Wang B., Yang B., Tang M.X., Dong X.Z. (2014). Use of Electrical Impedance Tomography to Monitor Regional Cerebral Edema during Clinical Dehydration Treatment. PLoS ONE.

[89] Shi Y., Tian Z., Wang M. Simultaneous Imaging of Intracerebral Hemorrhage and Secondary Ischemia with Electrical Impedance Tomography. Proceedings of the 2021 IEEE International Conference on Consumer Electronics and Computer Engineering, ICCECE 2021.

[90] Ferraioli F., Formisano A., Martone R. (2009). Effective Exploitation of Prior Information in Electrical Impedance Tomography for Thermal Monitoring of Hyperthermia Treatments. IEEE Trans. Magn..

[91] Bottiglieri A., Dunne E., McDermott B., Cavagnaro M., Porter E., Farina L. Monitoring Microwave Thermal Ablation Using Electrical Impedance Tomography: An Experimental Feasibility Study. Proceedings of the 14th European Conference on Antennas and Propagation, EuCAP 2020.

[92] Kao T.J., Boverman G., Kim B.S., Isaacson D., Saulnier G.J., Newell J.C., Choi M.H., Moore R.H., Kopans D.B. (2008). Regional Admittivity Spectra with Tomosynthesis Images for Breast Cancer Detection: Preliminary Patient Study. IEEE Trans. Med. Imaging.

[93] Akhtari-Zavare M., Latiff L.A. (2015). Electrical Impedance Tomography as a Primary Screening Technique for Breast Cancer Detection. Asian Pacific J. Cancer Prev..

[94] Mansouri S., Chabchoub S., Alharbi Y., Alshrouf A. (2022). EIT 40-Electrodes Breast Cancer Detection and Screening. IEEJ Trans. Electr. Electron. Eng..

[95] Ma G., Soleimani M. (2020). Spectral Capacitively Coupled Electrical Resistivity Tomography for Breast Cancer Detection. IEEE Access.

[96] Braun R.P., Mangana J., Goldinger S., French L., Dummer R., Marghoob A.A. (2017). Electrical Impedance Spectroscopy in Skin Cancer Diagnosis. Dermatol. Clin..

[97] Stojadinovic A., Fields S.I., Shriver C.D., Lenington S., Ginor R., Peoples G.E., Burch H.B., Peretz T., Freund H.R., Nissan A. (2005). Electrical Impedance Scanning of Thyroid Nodules before Thyroid Surgery: A Prospective Study. Ann. Surg. Oncol..

[98] Laufer S., Ivorra A., Reuter V.E., Rubinsky B., Solomon S.B. (2010). Electrical Impedance Characterization of Normal and Cancerous Human Hepatic Tissue. Physiol. Meas..

[99] Das L., Das S., Chatterjee J. (2015). Electrical Bioimpedance Analysis: A New Method in Cervical Cancer Screening. J. Med. Eng..

[100] Zuluaga-Gomez J., Zerhouni N., Al Masry Z., Devalland C., Varnier C. (2019). A Survey of Breast Cancer Screening Techniques: Thermography and Electrical Impedance Tomography. J. Med. Eng. Technol..

[101] Pathiraja A.A., Pathiraja A.A., Weerakkody R.A., Weerakkody R.A., Von Roon A.C., Von Roon A.C., Ziprin P., Ziprin P., Bayford R., Bayford R. (2020). The Clinical Application of Electrical Impedance Technology in the Detection of Malignant Neoplasms: A Systematic Review. J. Transl. Med..

[102] Yang D., Gu C., Gu Y., Zhang X., Ge D., Zhang Y., Wang N., Zheng X., Wang H., Yang L. (2022). Electrical Impedance Analysis for Lung Cancer: A Prospective, Multicenter, Blind Validation Study. Front. Oncol..

[103] Ye G., Lim K.H., George R.T., Ybarra G.A., Joines W.T., Liu Q.H. (2008). 3D EIT for Breast Cancer Imaging: System, Measurements, and Reconstruction. Microw. Opt. Technol. Lett..

[104] Hong S., Lee J., Bae J., Yoo H.J. (2015). A 10.4 MW Electrical Impedance Tomography SoC for Portable Real-Time Lung Ventilation Monitoring System. IEEE J. Solid-State Circuits.

[105] Murphy E.K., Skinner J., Martucci M., Rutkove S.B., Halter R.J. (2019). Toward Electrical Impedance Tomography Coupled Ultrasound Imaging for Assessing Muscle Health. IEEE Trans. Med. Imaging.

[106] Iqbal S.M.A., Mahgoub I., Du E., Leavitt M.A., Asghar W. (2021). Advances in Healthcare Wearable Devices. npj Flex. Electron..

[107] Huang J.J., Hung Y.H., Wang J.J., Lin B.S. (2016). Design of Wearable and Wireless Electrical Impedance Tomography System. Meas. J. Int. Meas. Confed..

[108] Rapin M., Braun F., Adler A., Wacker J., Frerichs I., Vogt B., Chetelat O. (2019). Wearable Sensors for Frequency-Multiplexed EIT and Multilead ECG Data Acquisition. IEEE Trans. Biomed. Eng..

[109] Zlochiver S., Arad M., Radai M.M., Barak-Shinar D., Krief H., Engelman T., Ben-Yehuda R., Adunsky A., Abboud S. (2007). A Portable Bio-Impedance System for Monitoring Lung Resistivity. Med. Eng. Phys..

[110] Wu Y., Jiang D., Duan J., Liu X., Bayford R., Demosthenous A. Towards a High Accuracy Wearable Hand Gesture Recognition System Using EIT. Proceedings of the IEEE International Symposium on Circuits and Systems.

[111] Lu X., Sun S., Liu K., Sun J., Xu L. (2022). Development of a Wearable Gesture Recognition System Based on Two-Terminal Electrical Impedance Tomography. IEEE J. Biomed. Health Inform..

[112] Lee M.H., Jang G.Y., Kim Y.E., Yoo P.J., Wi H., Oh T.I., Woo E.J. (2018). Portable Multi-Parameter Electrical Impedance Tomography for Sleep Apnea and Hypoventilation Monitoring: Feasibility Study. Physiol. Meas..

[113] Aliverti A. (2017). Wearable Technology: Role in Respiratory Health and Disease. Breathe.

[114] Angelucci A., Aliverti A. (2020). Telemonitoring Systems for Respiratory Patients: Technological Aspects. Pulmonology.

[115] Angelucci A., Cavicchioli M., Cintorrino I.A., Lauricella G., Rossi C., Strati S., Aliverti A. (2021). Smart Textiles and Sensorized Garments for Physiological Monitoring: A Review of Available Solutions and Techniques. Sensors.

[116] Hong S., Lee J., Yoo H.J. Wearable Lung-Health Monitoring System with Electrical Impedance Tomography. Proceedings of the Annual International Conference of the IEEE Engineering in Medicine and Biology Society, EMBC.

[117] Adler A., Grychtol B., Bayford R. (2015). Why Is EIT so Hard, and What Are We Doing about It?. Physiol. Meas..

